# Percutaneous Treatment of Hydatid Cysts with the Örmeci Technique

**DOI:** 10.5152/tjg.2024.23286

**Published:** 2024-05-01

**Authors:** Necati Örmeci, Özgün Ömer Asiller, Ayhan Parmaksız, , Çağdaş Kalkan, Evren Üstüner, Ahmet Altınel, Hakan Erdem

**Affiliations:** 1 Department of Internal Medicine, İstanbul Health and Technology University, İstanbul, Türkiye; 2 Department of Anesthesiology and Reanimation, Ankara University Faculty of Medicine, Ankara, Türkiye; 3 Department of Biostatistics, İstanbul Health and Technology University, İstanbul, Türkiye; 4 Ministry of Health, Ankara City Hospital, Ankara, Türkiye; 5 Department of Radiology, Ankara University Faculty of Medicine, Ankara, Türkiye; 6 Department of Infectious Diseases and Clinical Microbiology, Turkish Health Sciences University, Gülhane Faculty of Medicine, Ankara, Türkiye; 7 ID-IRI Lead Coordinator, Ankara, Türkiye

**Keywords:** Hydatid cyst, percutaneous treatment, Örmeci technique

## Abstract

**Background/Aims:**

The percutaneous route is accepted as the most convenient course in the management of hydatid cysts (HCs). The aim of this study is to analyze the efficacy of the Örmeci technique used in the treatment of hydatid cysts.

**Materials and Methods:**

This is a retrospective cohort study. Patients with HCs who presented to the Ankara University Faculty of Medicine, Department of Gastroenterology since 1991 were included. Patients with World Health Organization cystic echinococcosis (WHO-CE) types 1, 2, 3A-3B live cysts who were treated percutaneously at least once and followed up after a minimum of 6 months were analyzed.

**Results:**

A total of 1556 cystic lesions in 1035 patients have been presented to our department since 1991. Five hundred forty-four live HCs in 479 patients were treated with the Örmeci technique. The mean follow-up time was 59.29 months for females and 57.18 for males. The overall clinical success rate of all treated cysts with the Örmeci technique was 94.5%. After the treatment, a statistically significant decrease was found in all WHO-CE cyst types in terms of cyst sizes (*P* < .001 for all). Mortality, abscess and fistula formation, sclerosing cholangitis, and drug toxicities were not detected. Only 2 patients experienced reversible anaphylaxis during the treatment among 544 cysts (0.36%).

**Conclusion:**

Hydatid cysts can be treated percutaneously by the Örmeci technique with a high success rate for WHO-CE type 1, 2, and 3B. The Örmeci technique is an economic, simple, cheap, and repeatable outpatient procedure. It can be chosen as the first-line therapeutic modality in suitable patients with HCs.

**Main Points**

- The Örmeci technique has unique differences that make it more successful when compared to the puncture, aspiration, injection, reaspiration (PAIR) technique.
- Although the clinical treatment success was 88.18% for World Health Oganization cystic echinococcosis type 2 and 3B hydatid cysts, it was even higher in our technique than the success rates of PAIR technique and surgery according to published articles.
- The Örmeci technique has no mortality, and fewer morbidity.
- The Örmeci technique is safe, successful, cheap, and easy to use. It should be the first choice in the treatment of uncomplicated hydatid cysts.

## Introduction

Hydatid disease (HD) is caused by the larval stage of the *Echinococcus granulosus* (EG) and can cause cysts anywhere in the human body. It is still an important health and economic problem in endemic areas. The prevalence of EG infections is reported to be 1-200/100.000 in Türkiye, Romania, and Bulgaria with a worldwide distribution, and it affects an estimated 1.2 million people, particularly in pastoral communities. In addition, the total cost of hospitalization for hydatid cyst (HC) patients, including hospital stays and surgical interventions, exerts significant pressure on the healthcare system.^1,2^

The optimization of therapy for HC has been at the core of long debates over the years. Medical, surgical, and percutaneous treatment modalities have been used, but with high therapeutic failure rates.^3-6^ Treatment with antiparasitic drugs like mebendazole or albendazole is not the ideal treatment for HD, and the therapeutic failure is high with medical treatment. Although radical surgery was the gold standard 3-4 decades ago and has proven to decrease recurrence rates, it carries a high risk of intraoperative complications like bleeding and high rates of mortality and morbidity.^7^ Though laparoscopic surgery is effective in selected cases, particularly for the anterior cysts, increased risks for anaphylaxis and spillage and difficulties in laparoscopy maneuvers are known to exist. Accordingly, the puncture of the HC has long been contraindicated to avoid rupture that can lead to anaphylaxis and seeding along the puncture route. However, treatment policy began to change after the 1980s, and percutaneous treatment became an alternative to surgery during the last 2 decades.^8^ Today, the percutaneous route is accepted as the most convenient course in the management of HCs.^9^

Hence, the aim of this paper is to present our results in the percutaneous treatment of HCs using the Örmeci technique, which is a highly efficient and easy-to-use procedure compared to other percutaneous techniques.

## Materials and Methods

This is a retrospective cohort study.

### Setting

Patients with HCs admitted to the Ankara University Faculty of Medicine, Department of Gastroenterology with live HCs have been treated with the Örmeci technique since 1991. The most recent approval date was July 3, 2021.

### Design

The patients with World Health Organization cystic echinococcosis (WHO-CE) types 1, 2, 3A-3B live cysts who had been treated percutaneously with the Örmeci technique once at the minimum and followed up after at least 6 months were evaluated for the efficacy of treatment results. Most of the patients with HCs were diagnosed by abdominal ultrasonography. The HC diagnosis was based upon Gharbi or WHO classifications.^10,11^ In cases where the diagnosis was uncertain, a hemagglutination inhibition test, abdominal computed tomography, and/or magnetic resonance imaging were also requested. All patients were checked with blood tests such as Alanine Aminotransferase (ALT), Aspartate Aminotransferase (AST), Gama glutamic transpeptidase (GGT), alkaline phosphatase, bilirubin, and prothrombin time preceding the procedure.

### Inclusion Criteria

All live HCs, WHO-CE type1, type 2, type 3A, and type 3B, higher than 4 cm in diameter, were included for percutaneous treatment with our technique.

### Exclusion Criteria

Patients who refused to provide consent, those with cysts smaller than 4 cm in diameter, and patients with dead HCs (WHO-CE types 4 and 5) were not treated with the Örmeci technique and were excluded from the study.

### Data Collection

The data of HC patients have been retrieved from patients’ files stored by the first author since 1991. Hence, when there were missing data or inconsistencies in the database, the original patient files were checked in detail to provide a robust database.

### Örmeci Technique

All procedures were performed under sonographic guidance in the ultrasonography unit of a gastroenterology department that was fully equipped for an emergency condition, and all patients were initially treated on an outpatient basis. An *intra venous (i.v.) *line was established and 50 mg of meperidine, 40 mg of methyl prednisolon, pantaprasole 40 mg were given 20 minutes before the procedure. After sedo-analgesia, the percutaneous puncture was performed under sonographic guidance by using a 22-gauge Chiba needle as a 1-step procedure. To avoid spillage of cyst contents into the peritoneal cavity, the longest distance to the cyst was selected so as to leave a normal liver parenchyma between the cyst and liver capsule.

In the puncture, aspiration, injection, reaspiration (PAIR) technique, the HC is punctured, the drainage catheter is placed into the cyst and all of the fluid of the cysts is aspirated for WHO CE-type 1, type 2, and type 3A. The cyst cavity is filled with pure alcohol or hypertonic (20%) serum. After half an hour, all of the fluid of the cyst is re-aspirated. Aspiration of the solid part of WHO-CE type 3B cysts is difficult and the success rate is less than WHO CE type 1, type 2, and type 3A.

Unlike the PAIR technique, our procedure includes puncture of the cyst, 3 cm^3^ cyst juice for each centimeter of the long diameter of the cyst is aspirated. Then, for each centimeter of the long diameter of the cyst, 2 cm^3^ of absolute alcohol and 1 cm^3^ of polidocanol (aethoxysklerol 1%, Kreussler Pharma, Wiesbaden, Germany) is injected into the cyst cavity. One of the advantages of our technique is also to treat WHO-CE type 2 and type 3B cysts. WHO-CE type 2 and type 3B cysts were punctured by 3-5 different directions at the same session. Then, absolute alcohol and polidocanol were injected 3 cm^3^ for each centimeter of the long diameter of the cyst without aspiration. Polidocanol was chosen as a sclerosing agent to destroy the germinative membrane of the cyst, to enhance the sclerosing effect of absolute alcohol, and to obstruct the connection, if there is any, between the cyst cavity and biliary ducts or vessels. The needle was left 5 minutes inside the cyst within its sheath and then drawn back. The patient was observed for at least 6 hours. Then, if no adverse reaction had occurred, the patient was allowed to go home.^6^ The patients were followed up just 1 day after, 3 months after, 6 months after, and then every year.

The following findings were assigned as therapeutic successes during follow-up.

- Wholly The entire detachment of germinative membrane, particularly for WHO-CE type 1 and WHO-CE type 3A types.
- A decrease in cyst volume or the disappearance of the cyst occurred after the treatment.
- Solidification (1/3, 2/3, or total solidification) of the cyst; full calcification of the cyst
- The disappearance of daughter cysts

The parameters related to therapeutic failure were:

- Any cystic lesion on the puncture route
- Minimal or absence of change in cyst size
- Any activation of a dead cyst after percutaneous treatment.

### Interpretation of Imaging

**Figure 1.**
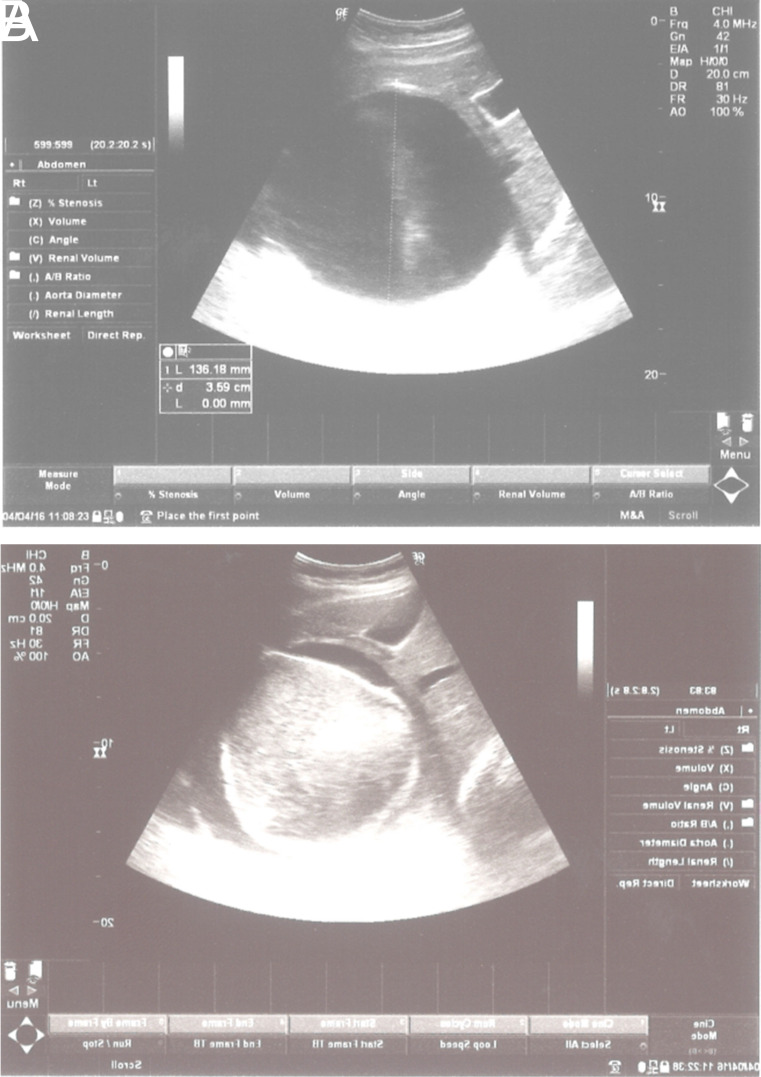
(A) WHO CE type 1 located in right lobe of the liver, before treatment. (B) The same cyst became like a snowball, the germinative membrane detached and turned white, was torn apart right after the injection of pure alcohol and polidocanol.

**Figure 2.**
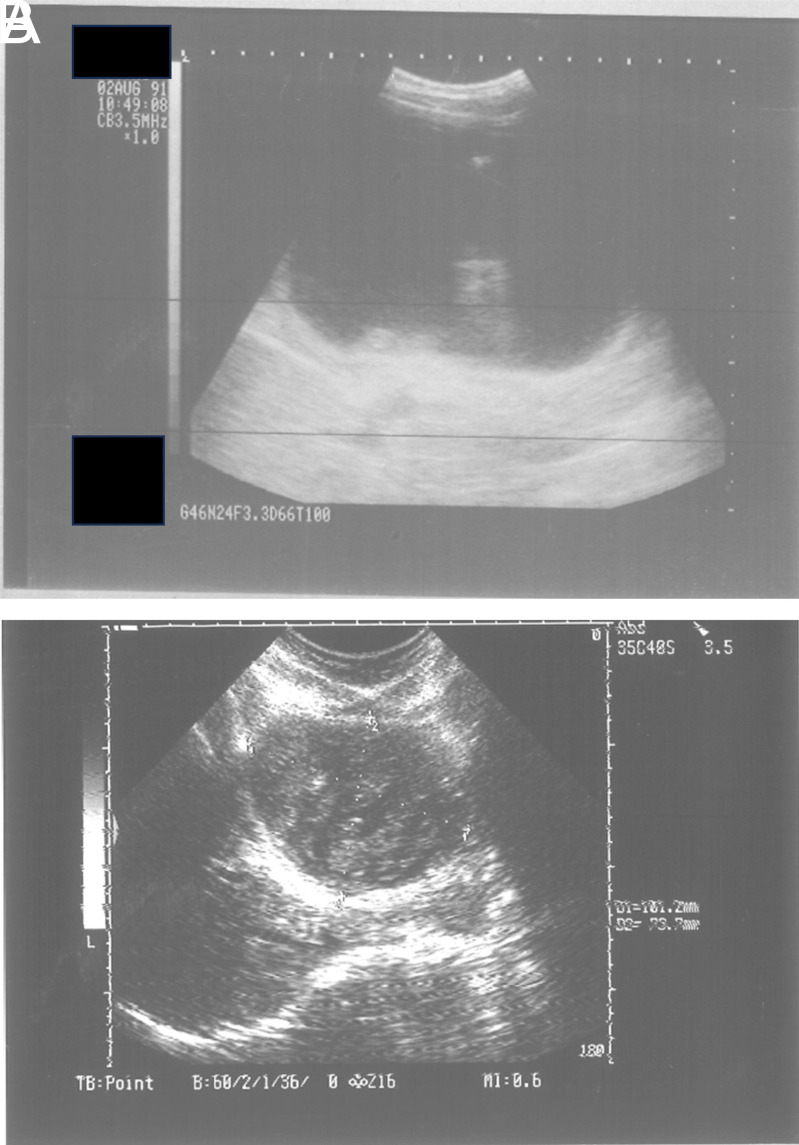
(A) WHO CE type 1 hydatid cyst located in right lobe of the liver. This is the first case treated on August, 1991. (B) The same cyst in panel A, totally solidified after the treatment.

**Figure 3.**
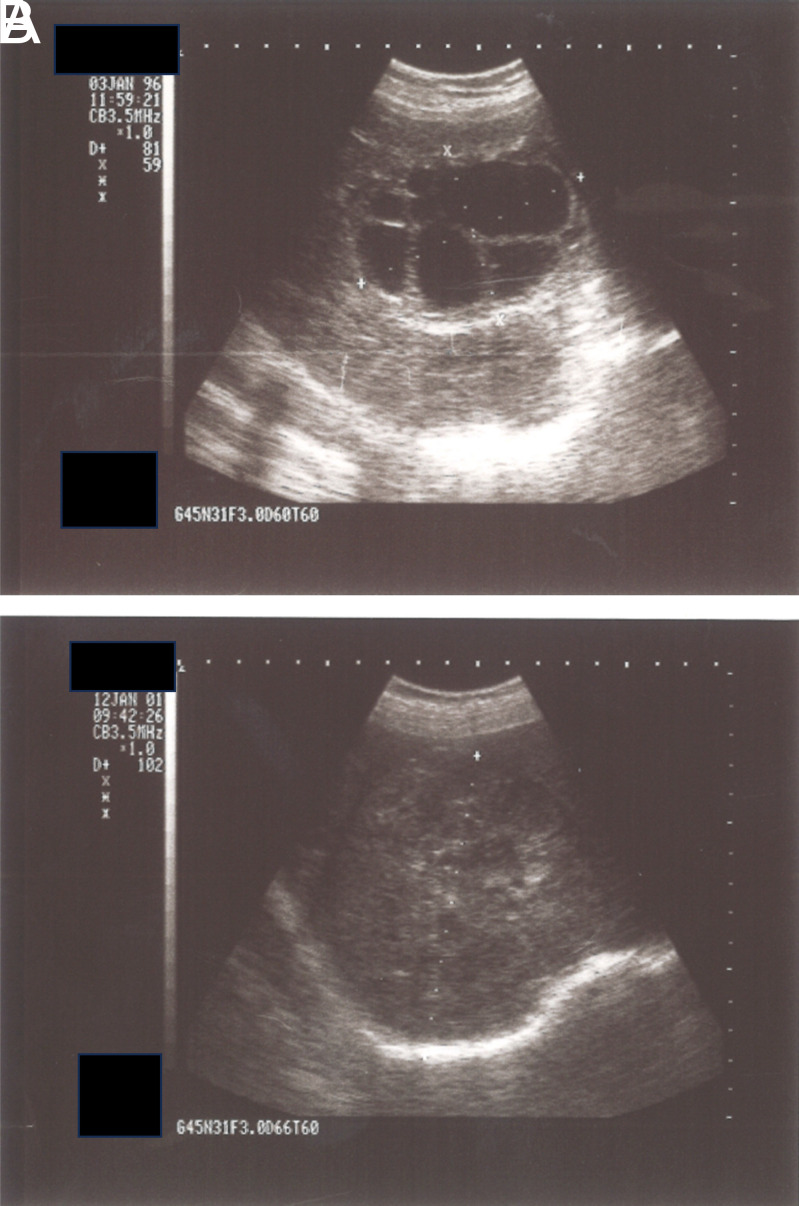
(A) WHO-CE type 2 hydatid cyst located in right lobe of the liver. (B) The same cyst in panel A, solidified after the treatment.

**Figure 4.**
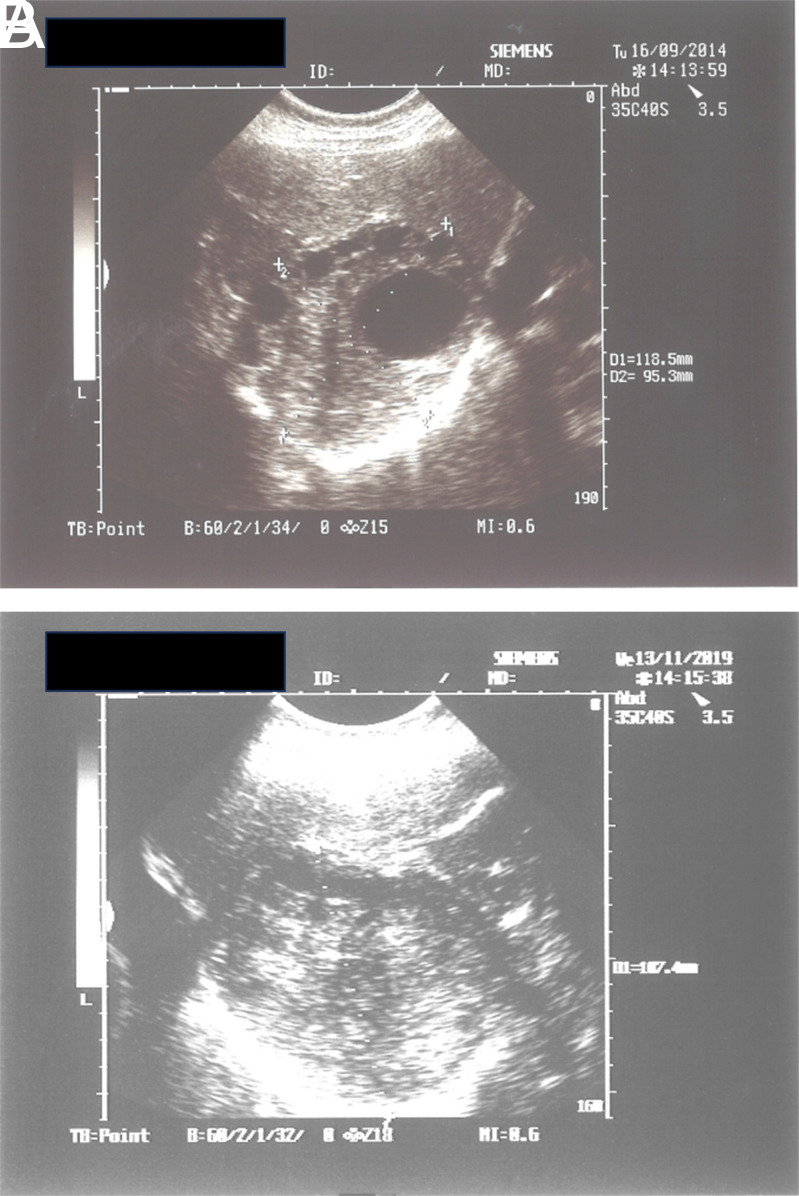
(A) WHO-CE type 3B hydatid cyst located in the right lobe of the liver. (B) The same cyst in panel A, solidified totally after the treatment.

**Figure 5.**
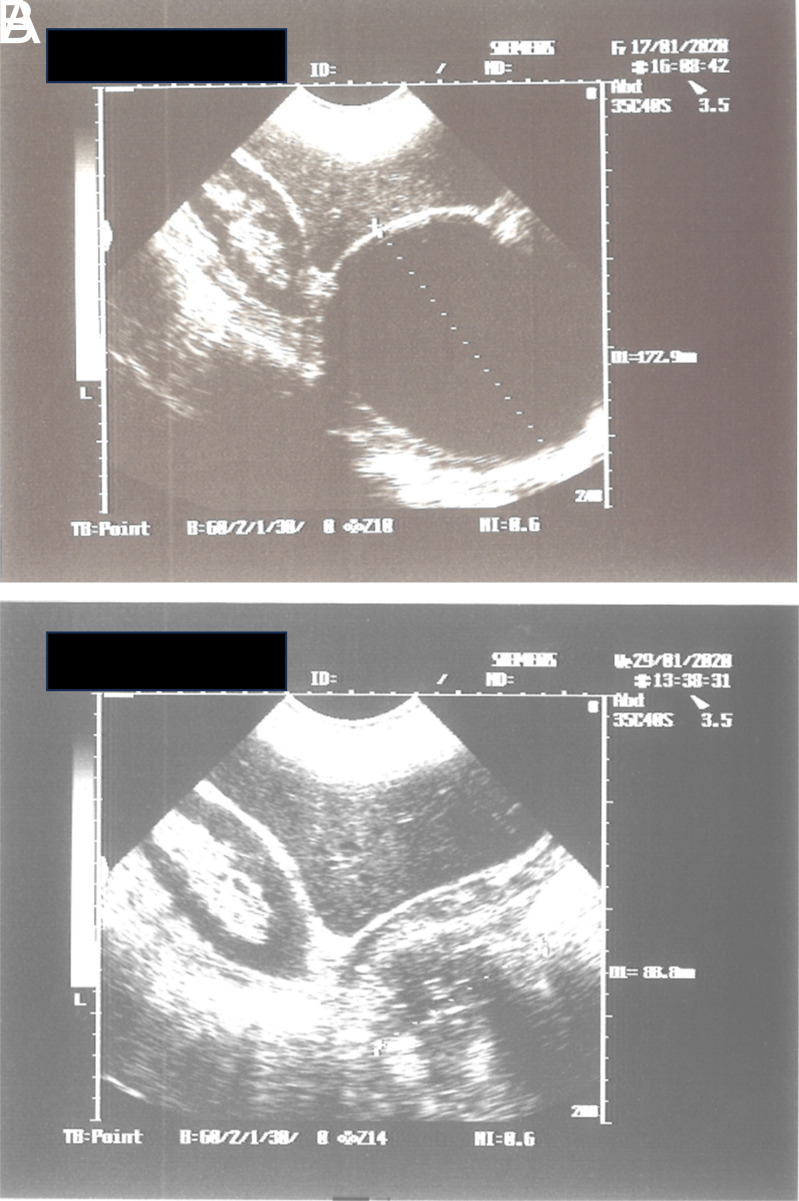
(A) A huge WHO-CE type 1 HC in the lung, extending to the liver and the vicinity of the right kidney, ruptured into the bronchus during the treatment. The cyst disappeared totally after the treatment. (B) The same cyst disappeared after the treatment.

After the treatment, WHO-CE type 1 cysts became like snowballs, germinative membranes were detached and turned white (Figure 1A and 1B) and were torn apart right after the injection of pure alcohol and polidocanol. WHO type 2 and/or type 3B cysts degenerated just after the treatment. During the follow-up, most of the cysts were solidified as shown in Figure 2A and 2B; Figure 3A and 3B; Figure 4A and 4B; Figure 5A and 5B.

### Statistical Analysis

Qualitative data were summarized using frequency and percentage, and quantitative data by mean and standard deviation. Comparisons of dependent 2 groups were made using dependent samples *t*-test for quantitative variables. Multiple logistic regression analysis was performed using the backward elimination (backward LR) method with the independent variables thought to influence the treatment outcome (success/failure). Statistical interpretations were interpreted at the 5% significance level. Statistical analyses were performed with the Statistical Package for Social Sciences (SPSS) version 26.0 (IBM Corp.; Armonk, NY, USA).

### Ethics Committee Approval

The study was conducted according to the principles of the Declaration of Helsinki’s “Ethical Principles for Medical Research Involving Human Subjects,” (June 1964, last revision in October 2013). The Ethical committee of Ankara University approved this study on October 26, 2015 with decision number: 16-689-15. All patients signed an additional informed consent before enrollment.

## Results

A total of 1556 cysts were detected in 1035 patients in our department since 1991. The locations, types, and treatment numbers of these cysts in the patients are presented (Table 1). In this study, the patients treated at least once and followed up after a minimum of 6 months were evaluated for the efficacy of treatment results for 544 cysts belonging to WHO-CE types 1, 2, 3A, and 3B live cysts in 479 patients. Five hundred fifty-six patients with 1012 HCs were not treated for reasons such as WHO type 4 or 5 dead cysts, cysts smaller than 4 cm in diameter, or patients having rejected the treatment. Two hundred ninety-nine females [mean age 43.79 ± 16.163 years (range 6-89)]; and a hundred seventy-eight male patients [mean age 43.99 ± 15.962 (range 6-75)] were treated by this technique. The mean follow-up time was 59.29 months for females and 57.18 for male patients.

### Distribution of the Cysts

Eighty-eight percent of the cysts (n = 485) treated were in the liver (right lobe: 70.05% (n = 384); left lobe 18.70% (n = 101), 5.26% (n = 28) in the spleen, 1.54% (n = 13) of in the muscles, 1.47% (n = 7) in unusual locations, and 1.22 % (n = 7) in the peritoneum. The cysts were more than 1 in 0.89% of the cases.

### Therapeutic Success

The comparison of cyst types before and after treatment is shown in Table 2. According to the post-treatment assessment, intervention was accepted as successful [type 0 (disappeared cysts) (n = 21), WHO-CE type 4 or WHO-CE type 5 in 257 (96.98%) of 265 WHO-CE type 1 cysts, in 56 (94.92%) of 59 WHO-CE type 3A cysts, and in 194 (88.18%) of 220 WHO-CE type 2 and 3B cysts. After the treatment, a statistically significant decrease was found for all WHO-CE cyst types (type 1, type 2, and type 3) in terms of cyst sizes (Table 2). The treatment successes recorded in post-treatment assessments are shown in Table 3.

### Multiple Logistic Regression Analysis

In the first step, age, gender, the size of cyst (size 1 × size 2), the type of cyst, and the location of cyst were included in the model (Table-S1). At the end of the fifth step, statistically significant associations were found between the risk of failure and the type of cysts. The risk of failure in WHO-CE types 2 and 3B group was 3.07 times higher than in WHO-CE type 1 group (odds ratio = 3.067, 95% CI, 1.316-7.151, *P* = .009). Five hundred fourteen cysts (94.5%) were successfully cured after the treatment while treatment failed in 30 cysts. The main therapeutic differences between the Örmeci technique and PAIR technique are seen in Table 4.

### Adverse Effects

A couple of complications were recorded in our patients after the percutaneous treatment (Table 3). There was no difference among the cyst types in terms of overall post-treatment complications [cyst type 1 (n = 11, 4.2%); cyst type 3A (n = 3, 5.1%); cyst types 2 and 3B (n = 7, 3.2%)] (*χ*^2^ = 0.572, *P* = .751).

## Discussion

The patients treated at least once and followed up after 6 months at the minimum were evaluated for the efficacy of treatment results for 544 cysts belonging to WHO-CE types 1, 2, 3A, and 3B live cysts in 479 patients. Overall, five hundred fourteen cysts (94.5%) were successfully cured after the treatment while treatment failed in thirty cysts. Although the clinical treatment success was 88.18% for WHO-CE type 2 and 3B HCs, it was higher in our technique than the success rates of the PAIR technique and of surgery according to published articles. The Örmeci technique has no mortality, and fewer morbidity. It is safe, successful, cheap, and easy to use. It should be the first choice in the treatment of uncomplicated HCs.

Surgical treatment of HD was the gold standard for 3-4 decades. The aims of surgical treatments are to inactivate the HCs, remove the germinative membrane, protect against recurrences and complications, eliminate the whole live component of the cyst, evacuate the cystic cavity, and treat the residual cavity of the cyst. Radical procedures include peri-cystectomy, hepatic resection or segmentectomy. Radical surgical treatments increase intraoperative complications such as bleeding and mortality risk while decreasing recurrence rate and morbidity.^7^ Conservative surgical treatments include unroofing associated with various procedures for the management of the residual cavity. Those treatments are safe and effective but morbidities such as abscess, fistula, and recurrence rates are high, and the hospital stay is longer.^3,7,12,13^ Among the conservative techniques, omentoplasty is the most favorable procedure to eliminate deep abscess.^7,14^ Mortality, morbidity, and recurrence rates vary between 0.7%-6.5%, 20.4%-53.8%, 3.8%-20%, respectively, which are dependent on size, number, location of cysts, surgical techniques, technical instruments, and available surgeons.^15-18^ The predictive factors which increase morbidity are large cysts above 9 cm in diameter, cysts located under the dome of the diaphragm, cysts which contain bile, cysts which are Gharbi type II, type III, type IV, and type V, ruptured into the peritoneum, biliary space, or pleural space, older age, and conservative surgical treatments.^15,16^

Laparoscopic surgery is effective in specially selected cases which are simple, uncomplicated, superficial, and in the anterior part of the liver. In a meta-analysis, it has been found that there is no superiority between laparoscopic surgery and open surgery in terms of effectiveness, safety, mortality, or morbidity between the 2 surgical techniques in uncomplicated HCs. There are several advantages of laparoscopic surgery, such as less morbidity and short hospital stays compared to open surgery.^19^ Intra-parenchymal and posteriorly located deep cysts, multiple cysts, calcified cysts, and recurring cysts are difficult to treat laparoscopically. There are also some disadvantages, such as an increased risk of anaphylaxis, increased risk of spillage, and difficulties in executing several maneuvers.^20^

In this study, the Örmeci technique has been used for the percutaneous treatment of HCs located in the liver, the spleen, the muscles, the kidneys, the subepidermal area of the skin, and the peritoneum.^6,21-24^ for more than 3 decades. The overall clinical success with the Örmeci technique in our center was 94.5%. The risk of failure in WHO-CE type 2 with multivesicular and multiseptated appearance, and in WHO-CE type 3B HCs presenting as unilocular with daughter cysts and echoic areas inside was threefold higher than in WHO-CE type 1 group. Kabaalioğlu^25^ and Giorgio^26^ reported their success rate as 39% and 41.4% for WHO-CE type 2 and 3B HCs. Hence, although the clinical treatment success was 88.18% for WHO-CE type 2 and 3B HCs, it was higher in our technique than the success rates of PAIR technique and surgery.^7,12^ In addition, the Örmeci technique, as an easy and repeatable modality, resulted in an insignificant risk of complications compared to other therapeutic modalities, supported by our data.

One of the basic conveniences of the Örmeci technique is the availability of access for more than one puncture in accordance with the sizes and locations of daughter cysts distributed in the mother WHO-CE type 2 and type 3B cysts. Hence, pure alcohol and polidocanol diffuse efficiently inside the cysts, causing better sclerosing activity. We do not place a catheter in our technique. A single puncture performed in the PAIR technique may not distribute the sclerosing agents into the daughter cysts adequately, and thus, the risk of therapeutic failure apparently increases. Polidocanol (1%) was used for the first time in the Örmeci technique to close the connections between the cysts and vasculature and/or biliary ducts. It seems that the use of polidocanol (1%) contributes an additive effect on the sclerosing activity of alcohol and blocks leakage.^27^ Hence, we do not use chemotherapy before and after the treatment. In addition, a small amount of sclerosing agents, including pure alcohol and polidocanol 1%, is injected into the cyst cavity, unlike the PAIR technique. Aspirating a small amount of cyst fluid keeps intra-cystic pressure in balance and inhibits the development of cysto-biliary fistula. This may have contributed to the reduced morbidity and ease of use of the Örmeci technique. Hence, in our case series, 71 out of 544 cysts were treated 2 times, 9 cysts were treated 3 times, and 2 cysts were treated 4 times through the percutaneous route. Thus, another advantage of this technique is that it is repeatable in case of a treatment failure. The hospital stay is shorter than in the PAIR technique and surgical technique due to its outpatient basis procedure.

Golemanov et al^8^ treated 348 HCs with the PAIR technique and 24% of the patients had minor complications like fistula formation, urticaria, pain, and fluid collection in pleural space. In another study, 61 patients with HCs were successfully treated with the PAIR, and after 6 years of follow-up, 2 anaphylaxes, 2 abscesses, 1 fistula, 5 urticaria, and 6 patients had fever after the procedure (26%).^28^ Accordingly, Men et al^29^ treated 111 patients with the PAIR technique. Seven fistulae, 4 abscesses, 7 urticaria, and 1 febrile episode were seen as adverse effects (17%) and 3 cases (2.7%) had recurrences.^2^ We did not observe any abscesses and fistula formation, sclerosing cholangitis, or drug toxicities in the patient population treated with the Örmeci technique. Only 2 patients experienced reversible anaphylaxis during the treatment of 544 cysts (0.36%). The reversible anaphylaxis rate of our patients is less than 1.8% detected in percutaneously treated HCs reported by Neumayr et al.^30^ The low rate of anaphylaxis among our case series is likely due to the administration of prednisolone during the procedure. Our 2 patients with anaphylaxis were medically treated and are still alive. In addition, there was not a significant difference between the cyst types in terms of complication rate among our patients. Hence, the technique did not result in intolerable consequences.

Although the limitation of this study is its retrospective design, it would be almost impossible to enroll HD patients prospectively for such a rare disease. In conclusion, current guidelines indicate that patients with liver HCs should be assigned to therapeutic options based on the cyst stage, location, size, patient characteristics, the experience of the operator, and the capability of the healthcare setting. In that context, percutaneous treatment of HCs with the Örmeci technique is a safe, efficacious, repeatable, cheap, and an outpatient procedure. Anyone experienced in interventional ultrasonography is capable of learning and executing the technique.

## Figures and Tables

**Table 1.** The Locations, Types, and Treatment Numbers of Cysts That Were Diagnosed Since 1991

WHO-CE Cyst Type	No. of Tx	Left Lobe of Liver	Right Lobe of Liver	Spleen	Kidney	Peritoneum	Muscle	Multiple	Unusual	Total	Total Number of Tx
1	None	25	110	22	2	5	4	2	6	176	0
	1 time	66	283	23	2	3	7	0	3	387	387
	2 times	4	15	0	1	1	1	0	0	22	44
	3 times	0	1	0	0	0	1	0	0	2	6
3A	None	1	20	1	0	1	0	0	0	23	0
	1 time	15	68	0	0	0	0	0	0	83	83
	2 times	0	4	0	0	0	0	0	0	4	8
2 and 3B	None	29	55	5	0	2	0	1	2	94	0
	1 time	38	178	8	5	4	5	1	3	242	242
	2 times	12	26	3	0	0	2	0	2	45	90
	3 times	0	7	0	0	0	0	0	0	7	21
	4 times	1	1	0	0	0	0	0	0	2	8
4	None	81	239	18	0	2	4	4	0	348	0
5	None	8	56	2	0	1	0	0	0	67	0
8	None	11	27	0	3	0	0	6	7	54	0
Total		291	1090	82	13	19	24	14	23	1556	889

Tx, treatment.

**Table 2.** Pre–Post Treatment Assessments of the Cysts

Pre-treatment	Post-treatment					
Types of Cysts	0^a^	1	2	3	4	5
WHO-CE type 1 (n = 265)	14 (5.3)	6 (2.3)	1 (0.4)	1 (0.4)	204 (77)	39 (14.7)
WHO-CE type 3A (n = 59)	0 (0)	0 (0)	2 (3.4)	1 (1.7)	50 (84.7)	6 (10.2)
WHO-CE type 2 and 3B (n = 220)	7 (3.2)	0 (0)	0 (0)	19 (8.6)	154 (70)	40 (18.2)
Total	21 (3.9)	6 (1.1)	3 (0.6)	21 (3.9)	408 (75)	85 (15.6)
**Types of Cysts**		**Pre-Tx**	**Post-Tx**	***t*** **^b^**	***P***	***r***
Type 1 (n = 265)	Size 1	84.63 ± 2.4	54.98 ± 1.83	16.192	<.001	0.657
	Size 2	72.51 ± 2.16	46.92 ± 1.62	15.426	<.001	0.650
	Size 1 × size 2	7357 ± 462.11	3307.82 ± 208.1	10.952	<.001	0.625
Type 3A (n = 59)	Size 1	77.53 ± 3.73	55.37 ± 3.05	7.953	<.001	0.679
	Size 2	65.05 ± 3.44	47.22 ± 2.82	6.604	<.001	0.644
	Size 1 × size 2	5631.58 ± 543.34	3052.44 ± 365.68	7.395	<.001	0.773
Type 2 and 3B (n = 220)	Size 1	83.26 ± 2.24	62.36 ± 2.04	11.305	<.001	0.631
	Size 2	72.81 ± 2.12	54.92 ± 1.82	9.881	<.001	0.586
	Size 1 × size 2	7002.36 ± 435.54	4172.53 ± 264.62	8.613	<.001	0.658

Size 1 > size 2 in each cyst. Follow-up ≥ 6 months and number of treatments >0.

SE, standard error; Tx, treatment.

^a^Disappeared cysts after treatment(s).

^b^Dependent samples *t*-test.

**Table 3.** Outcome of Patients with Örmeci Technique

	Cyst Type 1 (n = 265)	Cyst Type 3A (n = 59)	Cyst Types 2 and 3B (n = 220)
Treatment Success			
Decreasing of cysts	211 (79.6)	40 (67.8)	144 (65.5)
Pseudo-solid pattern in one-third	64 (24.2)	20 (33.9)	14 (6.4)
Pseudo-solid pattern two-thirds	61 (23)	15 (25.4)	22 (10)
Pseudo-solid pattern total	113 (42.6)	21 (35.6)	155 (70.5)
Minimal changes	3 (1.1)	1 (1.7)	3 (1.4)
Calcification	39 (14.7)	6 (10.2)	39 (17.7)
Detachment of cysts membrane	165 (62.3)	39 (66.1)	34 (15.5)
Disappearance of the cysts	12 (4.5)	2 (3.4)	7 (3.2)
Complications			
Abscess	0	0	0
Anaphylaxis	1	0	1
AST or ALT increase	1	1	0
Direct/indirect bilirubin increase	0	0	0
Urticaria	2	1	0
Hypotensive attack	1	0	1
Fever	5	1	5
Rupture of the cyst into bronchus	1	0	0
Total	11 (4.2%)	3 (5.1%)	7 (3.2%)
*χ* ^2^	0.572		
*P*	.751		

**Table 4.** Main Differences of Treatment Between Puncture, Aspiration, Injection, Reaspiration Technique and Örmeci Technique

	PAIR	Örmeci Technique
Needle type	18-20 gauge	22 gauge
Amount of fluid aspirated	1/3-1/2 of cyst volume	12-40 mL
Sclerosants	Pure alcohol or hypertonic saline	Pure alcohol plus polidocanol
Drainage catheter	8-12 Fr catheter	No catheter
Time period	Until drainage is stopped spontaneously	5 minutes
Length of hospital stay	Long	Outpatient basis
Chemotherapy	Albendazole or praziquantel	No chemotherapy
Re-occurring treatment	Rare	As needed
Puncture number in 1 session	One puncture	1-5 punctures at the same time, in WHO-CE type 2 and/or type 3B

PAIR, puncture, aspiration, injection, reaspiration.
